# Effect of Dolutegravir on Plasma Glucose Among Human Immunodeficiency Virus Patients in a Community Health Center Setting

**DOI:** 10.7759/cureus.30556

**Published:** 2022-10-21

**Authors:** Foud Bahamdain

**Affiliations:** 1 Clinical Pharmacy, King Abdullah Medical City, Mecca, SAU

**Keywords:** combination antiretroviral therapy, pre-diabetes, dolutegravir, diabetes mellitis, hiv care

## Abstract

Background

Dolutegravir has become one of the initial backbones in antiretroviral therapy regimens for most patients with HIV in several recent clinical guidelines. However, dolutegravir has been associated with severe cases of hyperglycemia and the new-onset of diabetes in multiple case reports and clinical trials. A community health center noticed an increasing number of new-onset hyperglycemia incidences in patients on dolutegravir.

Method

Retrospective chart review of patients who started on dolutegravir or dolutegravir combination regimen (Triumreq®, Juluca®, GlaxoSmithKline (GSK), Research Triangle Park, NC) between the dates of January 1st, 2013 and January 1st, 2018 who have been treated in community healthcare centers. Baseline blood sugar and/or hemoglobin A1C before starting dolutegravir, at three to six months of treatment, and at the end of the study were compared between subjects. Four hundred twenty-two subjects were enrolled.

Results

Dolutegravir had little effect on plasma glucose among 72% of the subjects (n=305). However, 7% of the subjects (n=28) on dolutegravir treatment with no glucose intolerance met the criteria for prediabetes at three to six months of therapy. One of the subjects had developed diabetes three to six months after dolutegravir was initiated. In addition, at the end of the study, 13% of patients developed prediabetes (n=56) and 1.4% developed diabetes (n=6). Among the 24 subjects that had diabetes before dolutegravir was initiated, 83% required intensification of their diabetes regimen.

Conclusion

Dolutegravir may cause a moderate increase in plasma sugar after three to six months of therapy. Further increases in plasma sugar have been noticed in up to 13% of subjects meeting the criteria for prediabetes. Due to the existence of confounding variables, patients with diabetes should not be switched from dolutegravir.

## Introduction

Diabetes mellitus affects approximately 10% of the United States population and contributes to significant morbidity, decreased quality of life, rising health care costs, and mortality [1]. Research has demonstrated a four-fold increase in the incidence of diabetes in men with HIV infection taking antiretroviral therapy (ART) compared to men not infected with HIV [2]. In several recent clinical guidelines, integrase strand transfer inhibitor (INSTI)-based regimens are recommended as initial antiretroviral therapy (ART) for most patients with human immunodeficiency virus (HIV) infection [3]. Dolutegravir (DTG) is an integrase strand transfer inhibitor that has become an initial component in many antiretroviral therapy (ART) regimens. Dolutegravir is dosed once daily with a long half-life of nearly fourteen hours, does not require a pharmacokinetic booster, has high potency and barrier to resistance compared to some ART medications, and has minimum cytochrome P450 interactions compared to other ART medications [4]. The pharmacokinetic properties of dolutegravir have made it an appealing drug in the treatment of HIV. However, hyperglycemia has been reported as one of the potential side effects of dolutegravir (up to 11%) [5-7]. The severity of hyperglycemia ranged from grade 2 (plasma glucose level between 126 and 250 mg/dL) to grade 4 (a life-threatening complication like ketoacidosis) in several clinical trials that approved dolutegravir efficacy. Furthermore, dolutegravir has been associated with severe cases of hyperglycemia and new-onset of diabetes in multiple case reports [8-12]. The community health centers noticed an increasing number of new-onset hyperglycemia incidences in patients on dolutegravir. Due to the lack of clinical studies on the prevalence and association between new-onset of hyperglycemia and dolutegravir in a community health center setting, the need for a study that examined this phenomenon was necessary, as results can be used to guide providers in the selection of ART. The aim of this study is to investigate the association between dolutegravir and hyperglycemia and measure the impact of dolutegravir on patients with diabetes.

This research was presented as an abstract at the American Pharmacists Association (APhA2019), March 21-25, 2019, in Seattle [13].

## Materials and methods

This study was a retrospective chart review of patients who started on dolutegravir or a dolutegravir combination regimen (Triumreq®, Juluca®, GlaxoSmithKline (GSK), Research Triangle Park, NC) with or without the diagnosis of diabetes who had been treated in Tucson, Arizona, United States: El Rio Community Healthcare Centers between the dates of January 2013 and January 2018. We included participants who were 18 years or older with HIV infection and were on dolutegravir between January 2013 and January 2018. Patients were required to have at least one blood sugar and/or A1C, at three to six months after initiation of dolutegravir and at the end of the study or after dolutegravir was discontinued. We excluded individuals who were on protease inhibitors. Protease inhibitors acutely and reversibly inhibit the insulin-responsive glucose transporter Glut4, leading to peripheral insulin resistance and impaired glucose tolerance [11]. Children (age<17) and patients with documented pregnancy during the study period were also excluded.

The study was approved by the Research Oversight Committee (IRB) of El Rio Community Healthcare Centers and the University of Arizona, Tucson, Arizona, USA. The IRB and ethics committee waived the requirement for informed consent due to limited time and feasibility. Minors were excluded; thus, no consent was needed from parents or guardians. Patients were identified using medication prescription records. An electronic health record (EHR) was utilized to collect patients’ data using a data collection form. Baseline blood sugar and/or A1C before starting DTG, at three to six months of treatment, and at the end of the five-year study period were compared between groups. A further examination of how many patients in the non-diabetic group have developed diabetes mellitus or prediabetes was done. Finally, the number of patients in the diabetes group requiring escalation in therapy was identified. Only the average and percentage of patients were reported without the need for further analysis. 

## Results

**Figure 1:**
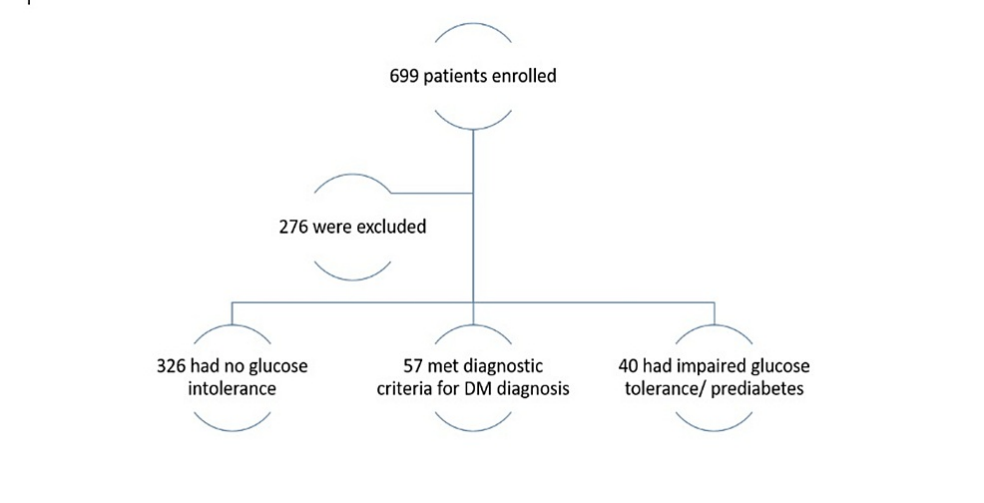
Study profile. DM: diabetes mellitus.

Between January 1st, 2013 and January 1st, 2018, 699 individuals were screened for inclusion, of which 423 were enrolled (Figure 1). Baseline characteristics are shown below (Table 1) and for the 423 subjects. The majority of subjects were male (85%). Forty-six percent of the subjects were of white/non-Hispanic heritage, with 36% of subjects being Hispanic/Latino and one percent of African American descent. Some patients (n=24) had diabetes before dolutegravir was initiated, and others had prediabetes (n=13). The majority of the subjects had chronic co-morbidities; defined as diagnoses of one of these diseases (diabetes, dyslipidemia, hypertension, polycystic ovary syndrome, any mental health disease, and a BMI of >24). Only 6% of the study population (n=24) did not have any chronic co-morbidities.

**Table 1:** Baseline characteristics and demographic data of patients.

Characteristic	Number of patients (%)
Male	358 (85)
Initial diagnosis of diabetes	24 (6)
Initial diagnosis of prediabetes	14 (3)
White/non-Hispanic	196 (46)
Hispanic/Latino	153 (36)
African American	43 (1)
Chronic co-morbidities	397 (94)

**Figure 2:**
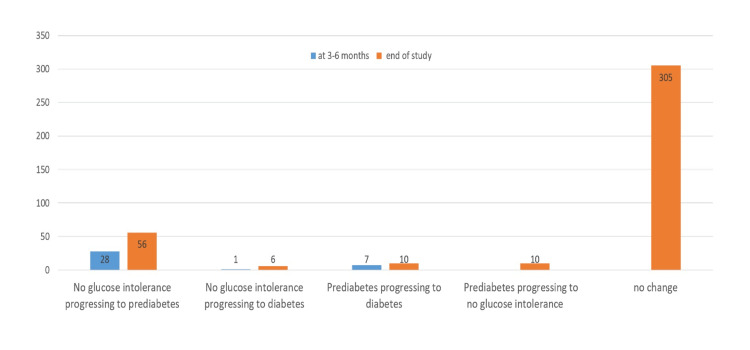
Effect of dolutegravir on plasma glucose after three to four months and at the end of the study. Data are in numbers (number of patients).

As shown in Figure 2, dolutegravir did not have any effect on plasma glucose at the end of the study among 72% of the subjects (n=305). However, 7% of the subjects (n=28) on dolutegravir treatment with no glucose intolerance met the criteria for prediabetes at three to six months of therapy. Moreover, one subject had developed diabetes three to six months after dolutegravir was initiated. In addition, at the end of the study, 13% of patients developed prediabetes (n=56) and 1.4% developed diabetes (n=6).

**Figure 3:**
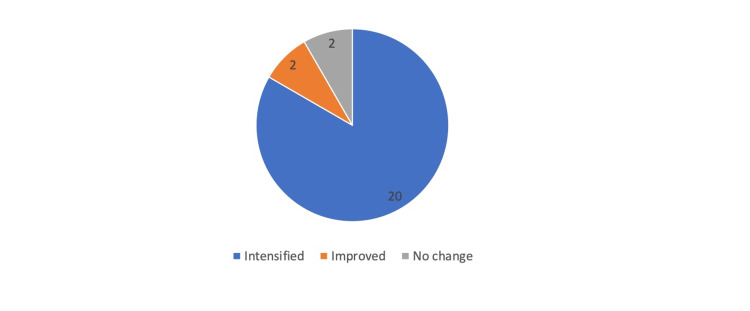
Number of patients that required intensification of their diabetes regimen.

Among the 24 subjects that had diabetes before dolutegravir was initiated, 83% required intensification of their diabetes regimen (Figure 3). Intensification of the diabetes regimen was defined as any increase in their current dose or the addition of new diabetes medication to the subjects’ regiment before dolutegravir was initiated.

## Discussion

Combination ART for the treatment of HIV infection is associated with a risk of developing diabetes, mainly in subjects with signs of metabolic syndrome before initiating ART [2]. Dolutegravir has become one of the initial backbones in antiretroviral therapy regimens for most patients with HIV in several recent clinical guidelines [3]. In this study, we examine the association between the new-onset of hyperglycemia and dolutegravir. 

While only 1.4% of subjects on dolutegravir treatment developed diabetes (n=6), 7% of the subjects (n=28) on dolutegravir treatment with no glucose intolerance met the criteria for prediabetes at three to six months. The number of subjects on dolutegravir treatment with no glucose intolerance who met the criteria for prediabetes increased to 13% (n=56) by the end of the study. This study had similar results to the SINGLE study [6]. In the SINGLE study, the dolutegravir/abacavir/lamivudine group had 9% in the grade 2 hyperglycemia classification (serum plasma glucose between 126 and 250 mg/dL) and 2% in the grade 3 classification (serum plasma glucose >25). Moreover, hyperglycemia is reported in SPRING-2, SAILING, SINGLE, and VIKING-3 [6-8]. Furthermore, the package insert for dolutegravir had information regarding plasma glucose abnormalities. A mechanism for the INSTI-induced hyperglycemia was hypothesized to be due to the chelation of magnesium, thereby inhibiting the release and signaling of insulin [14-18]. On the other hand, hyperglycemia was not reported in the original VIKING trial; however, it was included in the package insert [8]. This was the only large study that examined the effect of dolutegravir on blood glucose using both A1c and random blood glucose.

This study had several limitations. The study did not exclude patients with pancreatic cancer or chronic pancreatitis and patients on additional medication therapy known to cause hyperglycemia (e.g., antipsychotics). Moreover, the study was done in a small but diverse population, limiting the generalizability of the results. Baseline A1C and random blood glucose were not assessed due to a lack of data in addition to age and duration of medication. The majority of the subjects were white men. Additionally, the study did not measure medications using prescription fill history or viral load. Finally, 83% of the diabetic patients who required escalation in antidiabetic therapy could be due to economic, social, and behavioral factors. Besides that, diabetes is a progressive disease that could account for some of the diabetes medication escalations.

## Conclusions

Dolutegravir may cause a moderate increase in plasma sugar after three to six months of therapy that warrants escalation of therapy. Due to the existence of confounding variables and limited association analysis, patients with diabetes should not be switched from dolutegravir based on the result of this study. Especially, since INSTI is still commonly used as first-line therapy for the treatment of HIV. Further sub-analysis of this study should be done to examine patient predictors of dolutegravir-associated hyperglycemia. In addition, different dolutegravir regimens​ (e.g., Tivicay® vs Triumeq®, (GlaxoSmithKline (GSK), Research Triangle Park, NC)), the effect of other INSTIs on blood glucose, and the long-term effect of dolutegravir should be assessed.
